# Emergence and widespread circulation of a recombinant SARS-CoV-2 lineage in North America

**DOI:** 10.1016/j.chom.2022.06.010

**Published:** 2022-08-10

**Authors:** Bernardo Gutierrez, Hugo G. Castelán Sánchez, Darlan da Silva Candido, Ben Jackson, Shay Fleishon, Renaud Houzet, Christopher Ruis, Luis Delaye, Nuno R. Faria, Andrew Rambaut, Oliver G. Pybus, Marina Escalera-Zamudio

**Affiliations:** 1 Department of Zoology, University of Oxford, Oxford, UK; 2 Consorcio Mexicano de Vigilancia Genómica (CoViGen-Mex), México; 3 Colegio de Ciencias Biológicas y Ambientales, Universidad San Francisco de Quito USFQ, Quito, Ecuador; 4 Consejo Nacional de Ciencia y Tecnología, Ciudad de México, México; 5 Instituto de Medicina Tropical, Faculdade de Medicina da Universidade de São Paulo, São Paulo, Brazil; 6 Institute of Evolutionary Biology, University of Edinburgh, Edinburgh, UK; 7 Hertzl 52, Jerusalem, Israel; 8 10 rue de Dunkerque, 75010, Paris, France; 9 Molecular Immunity Unit, Department of Medicine, University of Cambridge, Cambridge, UK; 10 Department of Veterinary Medicine, University of Cambridge, Cambridge, UK; 11 Departamento de Ingeniería Genética, Unidad Irapuato, CINVESTAV, Irapuato, Mexico; 12 MRC Centre for Global Infectious Disease Analysis, School of Public Health, Imperial College London, London, UK; 13 The Abdul Latif Jameel Institute for Disease and Emergency Analytics, School of Public Health, Imperial College London, London, UK; 14 Department of Pathobiology, Royal Veterinary College, London, UK

**Keywords:** SARS-CoV-2, recombination, phylogenetics, molecular epidemiology

## Abstract

Although recombination is a feature of coronavirus evolution, previously detected recombinant lineages of SARS-CoV-2 have shown limited circulation thus far. Here, we present a detailed phylogenetic analysis of four SARS-CoV-2 lineages to investigate the possibility of virus recombination among them. Our analyses reveal well-supported phylogenetic differences between the Orf1ab region encoding viral non-structural proteins and the rest of the genome, including Spike (S) protein and remaining reading frames. By accounting for several deletions in NSP6, Orf3a, and S, we conclude that the B.1.628 major cluster, now designated as lineage XB, originated from a recombination event between viruses of B.1.631 and B.1.634 lineages. This scenario is supported by the spatiotemporal distribution of these lineages across the USA and Mexico during 2021, suggesting that the recombination event originated in this geographical region. This event raises important questions regarding the role and potential effects of recombination on SARS-CoV-2 evolution.

## Highlights

• Recombinant SARS-CoV-2 lineage (B.1.628/XB) circulated widely in North America in 2021
• Deletions in 3 loci verified recombination independently of phylogenetic analyses
• Epidemiological data suggest that XB emerged after high transmission in Mexico and USA

## Graphical abstract

**Figure:**
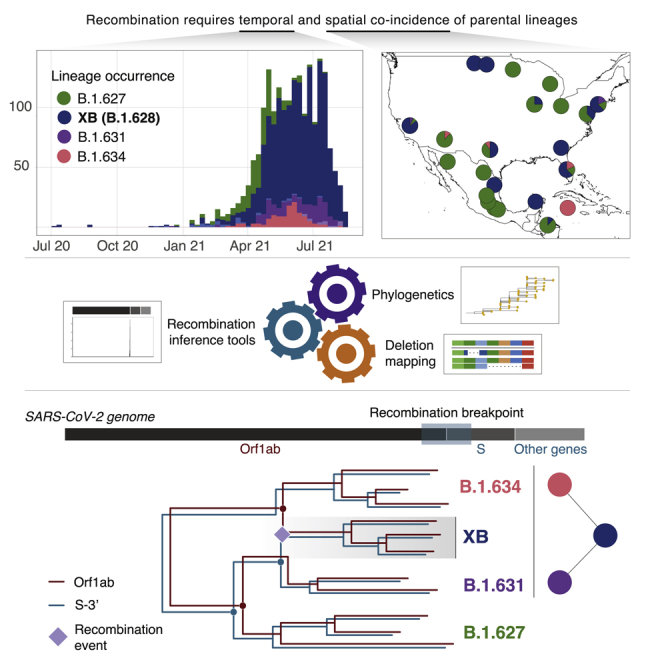

## Teaser

Gutierrez et al. identify the SARS-CoV-2 recombinant lineage XB. They use phylogenetics, recombination inference methods, and the tracking of deletions in the viral genome to identify its parental lineages. The size and spread of XB highlight the importance of identifying and tracking recombinant lineages as potential sources for new variants.

## Introduction

Virus recombination is the process by which genetic material from two genetically distinct parental lineages is combined into a viable descendant virus genome and is a common feature of sarbecovirus evolution (Boni et al., 2020). Genomic analyses suggest that recombination events among coronaviruses circulating in non-human species occurred during the evolutionary history of SARS-CoV-2 prior to its establishment in humans (Zhu et al., 2020; Li et al., 2020; Lytras et al., 2021). Signals of ongoing recombination among SARS-CoV-2 genomes have been assessed under a statistical framework during the COVID-19 pandemic (VanInsberghe et al., 2021). Most notably, viral genomes that are clearly recombinant have been observed at low frequencies in the UK, some of which showed evidence of forward transmission (Jackson et al., 2021). One of these UK recombinants was designated as lineage XA, the first recombinant lineage in the Pango nomenclature system (O’Toole et al., 2021; Rambaut et al., 2020). Potential recombinants between two variants of concern (VOCs), Alpha and Delta, have also been detected in a small cluster in Japan (Sekizuka et al., 2021), and more recently, another potential BA.1/BA.2 recombinant lineage has been identified in the UK and Ireland (reported in https://github.com/cov-lineages/pango-designation/issues/454). Although few clearly recombinant SARS-CoV-2 lineages have been reported so far, our ability to detect them is likely to increase as time progresses, given the continued genetic divergence of SARS-CoV-2 and the increased co-circulation of divergent lineages. Nonetheless, the detection of recombination within highly successful lineages with a limited genetic divergence will remain a challenge.

Our understanding of the effects of genomic recombination on SARS-CoV-2 fitness and transmission dynamics is still limited, but genetic exchange has been previously associated with evolutionary adaptation in viruses under experimental conditions (e.g., poliovirus; Xiao et al., 2016), in individual hosts (e.g., HIV; Song et al., 2018), and in nature (e.g., human influenza viruses; Petrova and Russell, 2018). Interestingly, a recombination event is associated with the emergence of a MERS-CoV lineage that became dominant in camels in the Middle East between 2014 and 2015 (Sabir et al., 2016). However, the question regarding the potential for recombination to contribute to SARS-CoV-2 evolution and adaptation remains. The emergence of highly divergent variants also raises questions regarding the role of recombination in the occurrence of large sequence shifts. Although there is currently no evidence suggesting that recombination played a role in the origins of the recently designated VOC Omicron (Pango lineage B.1.1.529) (Callaway, 2021; Technical Advisory Group on SARS-CoV-2 Virus Evolution, 2021), the accumulation of substantial numbers of mutations as observed in this variant could be produced by recombination mechanisms (Awadalla, 2003).

For virus recombination to occur, the parental lineages need to co-circulate in the same location to allow specific individuals to become co-infected. This scenario provides the circumstances during which chimeric genotypes can emerge, usually through molecular processes such as template switching, homologous recombination, or reassortment (the latter occurring in viruses with segmented genomes; Simon-Loriere and Holmes, 2011). Coronaviruses naturally produce a variety of recombination products during natural infection, including recombinant genomes, a process mediated by the proofreading exoribonuclease (Gribble et al., 2021).

Mosaic genomes likely resulting from recombination can be detected through changes in sequence similarities among different regions of the virus genome relative to parental lineages. Identifying recombination between recently diverged lineages is difficult because sequence similarity is high, and it is hard to distinguish homoplasic changes from those that are identical by descent due to inheritance from a recent shared ancestor (synapomorphic changes). In such instances, other mutations like insertions and deletions can prove informative; specifically, deletions are highly unlikely to revert during the evolution of a single lineage. Phylogenetic methods can also provide a tractable approach to test hypotheses regarding virus recombination, as they can be used to reconstruct the separate evolutionary histories of subgenomic regions (Simon-Loriere and Holmes, 2011). Although genome regions that share the same ancestry can be easily established for segmented viruses (e.g., Orthomyxoviruses, such as influenza viruses; Holmes et al., 2005), the exact start and endpoints of recombinant genome regions (namely, recombination breakpoints) must be inferred statistically for non-segmented viruses (Pérez-Losada et al., 2015). Furthermore, estimating the timing and location of recombination events can be limited by uncertainty in estimates of phylogenetic node ages, although such uncertainty can be reduced by using methods that combine evolutionary information across different genome regions (e.g., Raghwani et al., 2012).

As SARS-CoV-2 circulates around the world, new lineages emerge and are tracked using the Pango dynamic hierarchical nomenclature system (Rambaut et al., 2020). During late 2020 and early 2021, a series of lineages descending from B.1 were first detected in North and Central America. Specifically, lineages B.1.627, B.1.628, B.1.631, and B.1.634 were detected by the national genomic surveillance programs in the United States of America, Mexico, and other countries in the Americas, and their genomes were shared publicly on the GISAID database (Shu and McCauley, 2017). An unusually high number of genomic similarities were detected among these lineages, prompting the suggestion that recombination had occurred during their emergence and spread (first discussed on Twitter at https://twitter.com/babarlelephant/status/1425859582958653442 and on the Pango GitHub website at https://github.com/cov-lineages/pango-designation/issues/189). This hypothesis was based on a comparison of a mutational matrix for >40 distinct lineages co-circulating in the Americas (see STAR Methods) and from another preliminary analysis using a limited number of representative sequences (see the Virological post at https://virological.org/t/re-proposal-to-redesignate-b-1-631-as-recombinant-lineage-xb/746).

To formally investigate and test the hypothesis of recombination, we undertook an exhaustive analysis of all sequences available for each linage in question, using an appropriate and robust methodology. Here, we present the analysis of the spread and evolution of these four lineages and investigate the possibility that one or more recombination events contributed to their evolution. Our results provide evidence supporting a recombinant origin for lineage B.1.628 and its designation as a distinct recombinant lineage that presented forward transmission, circulating in multiple countries.

## Results

### Distribution of lineages B.1.627, B.1.628, B.1.631, and B.1.634

**Figure 1:**
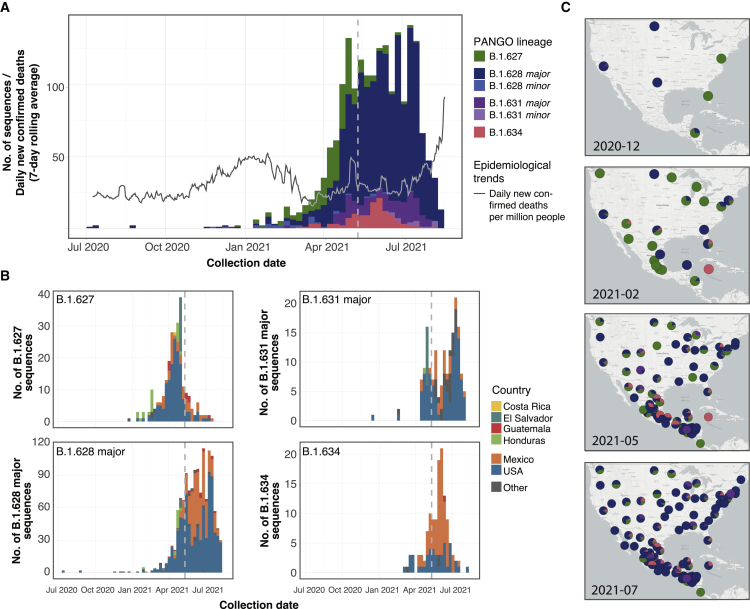
Spatiotemporal distribution of lineages B.1.627, B.1.628 (major and minor), B.1.631 (major and minor), and B.1.634 (A) Number of sequences on GISAID (per 2-week period, as of 2021-08-30) for each lineage publicly available in GISAID, plotted using the associated sample collection date for each sequence. The gray line shows the average number of new confirmed daily COVID-19-related deaths recorded in the North American region from July 2020 to July 2021 (data obtained from Our World in Data; Ritchie et al., 2020). (B) Weekly number of sequences for each lineage colored by country from where samples were collected. Countries outside of North and Central America make up <5% of sequences and are therefore grouped in the “Other” category. The dotted vertical lines show the starting of the systematic genome sampling and sequencing program according to the national SARS-CoV-2 genome surveillance program for Mexico (May 11, 2021). B.1.628 minor and B.1.631 minor lineages are not shown. (C) Mapping of the geographic spread of the four lineages in North and Central America (a region where >95% of the sequences were identified) at four representative months over their complete sampling date range.

Sequences for lineages B.1.627 (n = 252), B.1.628 (n = 1,391), B.1.631 (n = 181), and B.1.634 (n = 126) were collected between July 8, 2020 and August 18, 2021 (Table 1), however, the majority were sampled in 2021 (Figure 1A). All four lineages were predominantly sampled in North America (89.5% of sequences), either in the United States of America (USA) or Mexico. B.1.627 and B.1.631 were mostly sampled in the USA, whereas B.1.634 was most commonly found in Mexico (Figure 1B). Lineage B.1.628 is the most geographically widespread lineage in our dataset, identified in 41 different US states and in 31 Mexican states (all other lineages were identified in up to 21 US states and up to 17 Mexican states; Figure 1C). B.1.628 is also the most widely sampled through time, with 406 days between the earliest and most recent sample collection date (compared with B.1.627 = 212 days, B.1.631 = 232 days, and B.1.634 = 160 days). B.1.628 was sampled in the USA during the entirety of this sampling period, whereas it was only sampled for a period of 185 days in Mexico.

**Table 1:** Summary of sequences from four SARS-CoV-2 lineages associated with potential recombination (percentages shown in reference to the complete number of sequences in our dataset)

	**B.1.627**	**B.1.628**	**B.1.631**	**B.1.634**				
	N (%)	Date range	N (%)	Date range	N (%)	Date range	N (%)	Date range
**Africa**	0 (0%)	–	0 (0%)	–	20 (1.0%)	2021-05-21 to 2021-07-12	2 (0.1%)	2021-05-29
**Asia**	4 (0.1%)	2020-12-21 to 2021-02-28	16 (0.8%)	2020-11-15 to 2021-05-23	1 (0.05%)	2021-07-07	0 (0%)	–
**Caribbean Islands**	0 (0%)	–	3 (0.15%)	2021-06-02 to 2021-07-03	0 (0%)	–	1 (0.05%)	2021-03-11
**Central America**	46 (2.4%)	2021-01-04 to 2021-07-08	58 (3.0%)	2021-01-26 to 2021-07-26	11 (0.6%)	2021-04-23 to 2021-04-30	1 (0.05%)	2021-06-15
**Europe**	0 (0%)	–	24 (1.2%)	2021-01-04 to 2021-08-05	14 (0.7%)	2021-02-18 to 2021-08-05	0 (0%)	–
**North America**	202 (10.4%)	2021-01-16 to 2021-07-21	1,287 (66.0%)	2020-07-08 to 2021-08-18	134 (6.9%)	2020-12-16 to 2021-08-04	122 (6.3%)	2021-08-09
**Oceania**	0 (0%)	–	1 (0.05%)	2021-05-24	0 (0%)	–	0 (0%)	–
**South America**	0 (0%)	–	2 (0.1%)	2021-04-29 to 2021-08-03	1 (0.05%)	2021-05-28	0 (0%)	–
**TOTAL**	12.92%	2020-12-21 to 2021-07-21	71.33%	2020-07-08 to 2021-08-18	9.28%	2020-12-16 to 2021-08-05	6.46%	2021-03-02 to 2021-08-09

A maximum likelihood (ML) tree inferred from these genomes and rooted in the reference genome Wuhan-Hu-1 shows that all four lineages form monophyletic clusters as expected (Figure S1A). Two exceptions are noted for lineages B.1.628 and B.1.631. For the former, a group of sequences close to the root of the tree is designated as lineage B.1.628, and this group is distinct from the main B.1.628 clade. In the latter case, sequences from lineage B.1.631 are split into two paraphyletic clusters by B.1.627 (Figure S1A, inset). Results from UShER show similar patterns, with some B.1.628 and B.1.631 sequences clustering among other lineages (Figure S1B). UShER resolves the relationships between the four lineages under investigation sequentially, with B.1.627 diverging first, followed by B.1.634, and finally by B.1.631 and B.1.628. For reference purposes within this work, we henceforth refer to the larger monophyletic B.1.628 and B.1.631 clades as *B.1.628 major* and *B.1.631 major* and identify the sequences clustering at the base of the ML phylogeny (or among other B.1 lineages in UShER) that were assigned to these lineages as *B.1.628 minor* and *B.1.631 minor*. Some of the nodes that define important splits in the tree show moderate support, for example, the node that groups most B.1.631 genomes with other B.1.627 genomes (to the exclusion of the outlying B.1.631 genomes) is well supported (SH-aLRT = 98.6; Figure S1). The presence of these phylogenetic clusters that do not match the Pango lineage definitions (Rambaut et al., 2020) warrants further investigation, with recombination as a possible explanatory factor.

### Recombination analyses and breakpoint inference

**Figure 2:**
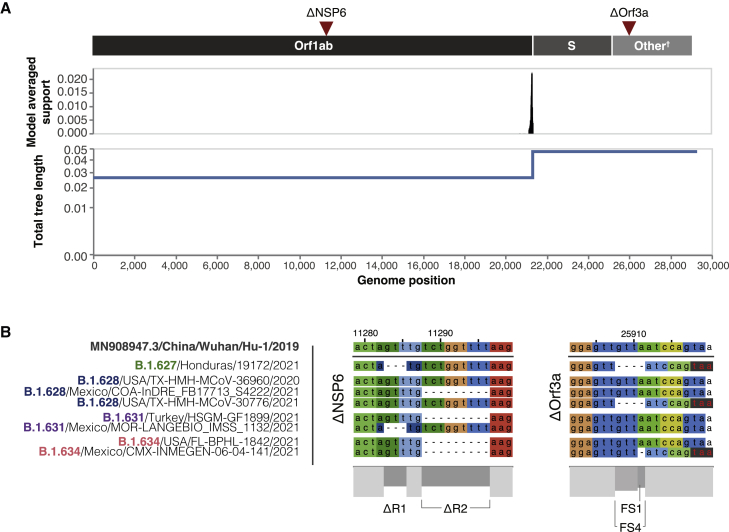
Recombination breakpoint analysis and deletions occurring in the four lineages (A) Recombination breakpoint analysis results performed on GARD show a statistically supported change in total tree length that stems from an inferred recombination breakpoint around the beginning of the S gene reading frame. The genomic location of deletions under investigation (ΔNSP6 and ΔOrf3a) are shown for reference. (B) Deletions in the NSP6 gene (Orf1ab) and Orf3a, illustrated on a representative selection of sequences that includes the B.1.627, B.1.628, B.1.631, and B.1.634 lineages. NSP6 deletions (ΔR1 and ΔR2) and Orf3a deletions (a single-nt frameshift deletion ΔFS2 or a 4-nt frameshift deletion (ΔFS4) are shown, with the early TAA stop codon produced by the Orf3a deletions shown in red letters on a black background.

Our results indicate that recombination is likely to have occurred. GARD analysis suggests that a single breakpoint in the alignment can explain the data (Figure 2A), with high support for a model incorporating this recombination event (Δc−AIC_*null model*_ = 202.176; Δc−AIC_*single tree, multiple partition model*_ = 565.199). The breakpoint inferred occurred around position 21,308 in reference to the Wuhan-Hu-1 genome (a TTT codon), corresponding to the signal peptide region at the N terminus of the spike protein (18 nt [nucelotides] downstream of the canonical sarbecovirus transcription regulatory sequence AACGAAC; Yang et al., 2021). However, some variation in the results was observed when using the subsampled datasets and when comparing the different methods used. For example, an independent analysis using GARD excluding the B.1.634 lineage revealed a recombination breakpoint inferred at position 22,775–22,778 (at a GAT codon) within the Spike protein reading frame (Δc−AIC_*null model*_ = 562.098; Δc−AIC_*single tree, multiple partition model*_ = 928.939). This corresponds to amino acid residue 390D located in the core region of the receptor-binding domain (RBD) adjacent to beta sheet 3 (β3; Lan et al., 2020). Moreover, the RDP4 results for the B.1.627 and B.1.628 sequences revealed a recombination breakpoint inferred at position ∼19,408/19,411 (*p*_MaxChi_ < 0.0001, *p*_3Seq_ < 0.0001), corresponding to the N7-MTase domain of nsp14, at the end of the Orf1ab. In any scenario, the receptor-binding motif (RBM) that includes the main ACE2 receptor contact points would have been inherited from the same parental lineage (B.1.628).

Recombination analysis outcomes also result in the placement of the NSP6 deletions on one side of the breakpoint and the Orf3a deletions on the other side of the breakpoint (Figures 2A and 2B). The NSP6 region contains two non-overlapping deletions: a 3-nt deletion (ΔR1) and a 6-nt deletion (ΔR2) that are 2 nt apart but do not derivate in frame shifts downstream (Figure 2B). The Orf3a displays two overlapping frameshift deletions: a single-nt deletion (FS1) and a 4-nt deletion (FS4) that overlap on the fourth position of FS4. Both deletions lead to the same early TAA/UAA termination codon, six nt downstream of the FS1/FS4 locus (Figure 2B).

### Phylogenetic discrepancies between non-recombinant genome segments

**Figure 3:**
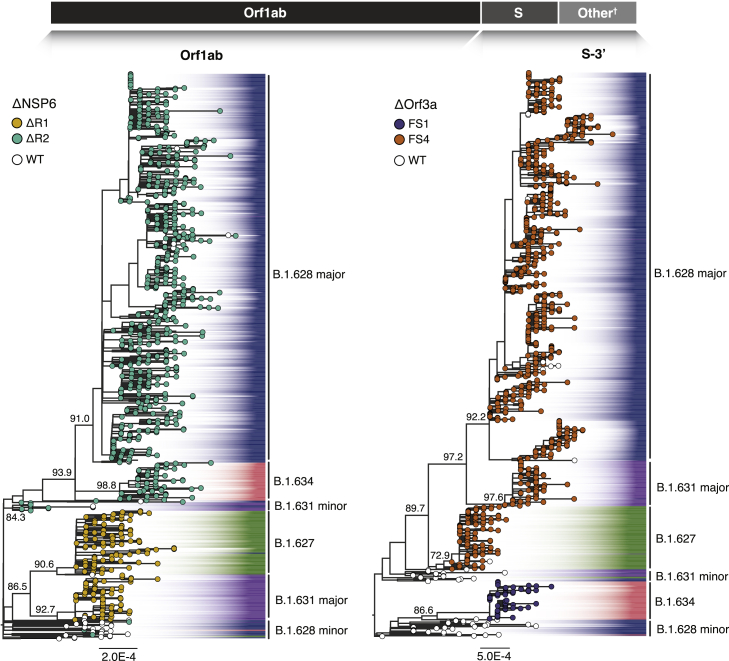
Maximum likelihood phylogenies of two segments of the SARS-CoV-2 genome for the four lineages Individual phylogenies were reconstructed for the 5′ and 3′ end of the viral genome (split at breakpoint 21,555–21,556), resulting in independent trees that represent the evolutionary history of Orf1ab (left) and of the S gene (plus the remaining structural and non-structural genes [other genes: E, M, N, Orf3a, Orf3b, Orf6, Orf7a, Orf7b, Orf8, Orf9b, Orf9c, and Orf10], referred here to as S-3′, right). The individually designated Pango lineages for each sequence are highlighted, whereas the consensus identifiers are also shown. For both trees, SH-aLRT support values are indicated for key nodes. Deletions occurring on each genome segment (ΔNSP6 on Orf1ab and ΔOrf3a on S-3′) are mapped onto the tips of the trees. For ΔNPS6 (S-3′ tree), tips displaying ΔR1 are shown in yellow, tips with ΔR2 in teal, and tips with no deletions (WT) in white. For ΔOrf3a (Orf1ab tree), tips displaying FS1 are shown in dark violet, tips with FS4 in orange, and tips with no deletions (WT) in white.

Given the inferred recombination breakpoint close to the start of the S reading frame (Figure 2A), we estimated separate phylogenetic trees for Orf1ab and for the remainder of the genome (including S and the remaining structural and non-structural genes, henceforth referred to as the S-3′ region). The phylogenies show topological discrepancies that coincide mostly with individual Pango lineages (Figure 3). Both phylogenies (rooted on the Wuhan-Hu-1 reference genome) show a poorly resolved early split, with Orf1ab showing a bifurcation into two monophyletic groups containing B.1.628 minor and B.1.631 minor basal sequences. On the other hand, the phylogeny for the S-3′ region places the B.1.628 minor sequences as a paraphyletic group from which B.1.634 descends (SH-aLRT = 91.9), while B.1.631 minor is a predecessor of the B.1.627, the B.1.628 major, and the B.1.631 major lineages (SH-aLRT = 79.7). On the Orf1ab phylogeny, lineages B.1.627 and B.1.631 major share a common ancestor (SH-aLRT = 86.5), whereas the S-3′ tree shows them as being paraphyletic. Lineage B.1.634 is consistently inferred as monophyletic in both trees: in the Orf1ab tree, it descends from B.1.631 minor, and in the S-3′ tree, from B.1.628 minor. The nodes defining lineages are generally well supported (SH-aLRT > 70.0), except for the basal nodes for the early bifurcation in both trees: statistical support within each lineage showed a combination of unsupported short branches (SH-aLRT = 0.0) and nodes with high support values (SH-aLRT > 75.0).

### Genome-wide divergence across genomes

To further explore the genetic divergence of these lineages and the sequences placed near the root of the trees (specifically, B.1.631 minor and B.1.628 minor), we estimated the pairwise genetic distances across the genomes of representative sequences (basal to the main clades) in reference to the Wuhan-Hu-1 reference genome (Figure S2). Although mutations have accumulated in all lineages, the Orf1ab region of B.1.631 minor shows the lowest divergence from Wuhan-Hu-1. All clades display peak genetic divergence between positions ∼21,000 and ∼23,000, with the exception of B.1.628 major, which diverges from Wuhan-Hu-1 homogeneously across its genome.

### Emergence of lineages from a B.1 background and recombination history

Given the genome-wide divergence observed for B.1.631 minor (particularly the unusually high similarities to Wuhan-Hu-1 in the Orf1ab region) and its limited spatiotemporal distribution (i.e., all sequences being from Turkey as opposed to the majority of the sequences that were observed in North and Central America), we excluded this group from further analyses. In particular, the fact that it was not observed anywhere in the Americas suggests that it would not have circulated in the same geographical region, a necessary condition for recombination to occur. In the absence of this cluster, we explored the evolutionary history of the remaining lineages in relation to other lineages that descend from B.1. A phylogenetic analysis including a sample from each lineage under investigation, B.1.1.7 (VOC Alpha) and B.1.351 (VOC Beta)—the two latter lineages were included in the analysis only for comparative purposes (see the STAR Methods section)—consistently shows that B.1.627, B.1.628 major, B.1.631 major, and B.1.634 group into well-supported monophyletic groups (bootstrap support >93%) for both the Orf1ab (Figure S3A) and the S-3′ (Figure S3B) genome segments, similar to the well-established Alpha and Beta VOCs. The B.1.628 minor sequences emerge from the polytomy that makes up the B.1 backbone and do not group with the B.1.628 major cluster, suggesting the former do not belong to the B.1.628 Pango lineage. Thus, sequences from B.1.628 minor were also excluded from further analyses. The Orf1ab phylogeny shows that B.1.627 and B.1.631 major share a common ancestor (bootstrap support = 62%), similar to B.1.628 major and B.1.634 (bootstrap support = 100%; Figure S3A)—this pattern is also observed in the full phylogeny (Figure 3). The S-3′ tree shows that B.1.628 major and B.1.631 major share a common ancestor (bootstrap support = 95%) which in turn descend from a common ancestor with B.1.627 (bootstrap support = 99%), whereas B.1.634 emerges independently from the B.1 background (Figure S3B); once again, the pattern is mirrored by the full S-3′ phylogeny (Figure 3). Based on the Pango system (and prior to this study), the minor and major clades identified here were initially assigned to either the B.1.628 or B.1.631 lineages. It should be noted, however, that the Pango lineage assignment is based on a machine learning approach that was not originally designed to consider recombination. Thus, it is not surprising that recombinant sequences will be miss-assigned until the program is re-trained with an updated designation of the recombinant sequences (O’Toole et al., 2021).

Reconciling the occurrence of the NSP6 and Orf3a deletions (Table S1) with the reconstruction of the evolutionary histories of these lineages for both genome segments is possible given that (1) none of the NSP6 deletions occurred simultaneously on the same sequence (it is either ΔR1 or ΔR2 in each sequence) and (2) the Orf3a deletions are overlapping (i.e., FS1 is contained within FS4). This makes it possible to encode ΔR1, ΔR2, FS1, and FS4 as unique traits and to map them to the phylogenetic trees (Figure 3). A third deletion, Δ69-70 on the S protein (also observed in previous VOCs and VOIs; Meng et al., 2021), was found to be exclusive to B.1.634 and therefore not used as an informative marker. From the Orf1ab tree, ΔR1 is shared between B.1.627 and B.1.631 major, whereas ΔR2 is shared by B.1.631 minor, B.1.628 major, and B.1.634. The most parsimonious explanation for this deletion pattern would suggest that ΔR1 occurred once (and predates the ancestral form of the Orf1ab of the B.1.627 and the B.1.631 major lineages) and that ΔR2 occurred once (predating the ancestral form of the Orf1ab for the B.1.628 major and B.1.634 lineages). Similarly, the two distinct frameshift deletions in Orf3a appear to have occurred independently: FS1 occurred once in the ancestral form of lineage B.1.634, and FS4 occurred at least once in the ancestral form of B.1.631 major, B.1.628 major, and B.1.627 (Figures 3 and4, upper). A considerable number of B.1.628 minor sequences in both trees (31/34 for Orf1ab, 31/31 for S-3′) share the wild-type trait (i.e., no deletion) with the Wuhan-Hu-1 reference genome, further suggesting that this group of sequences belongs to either B.1 or a different B.1.X lineage.

**Figure 4:**
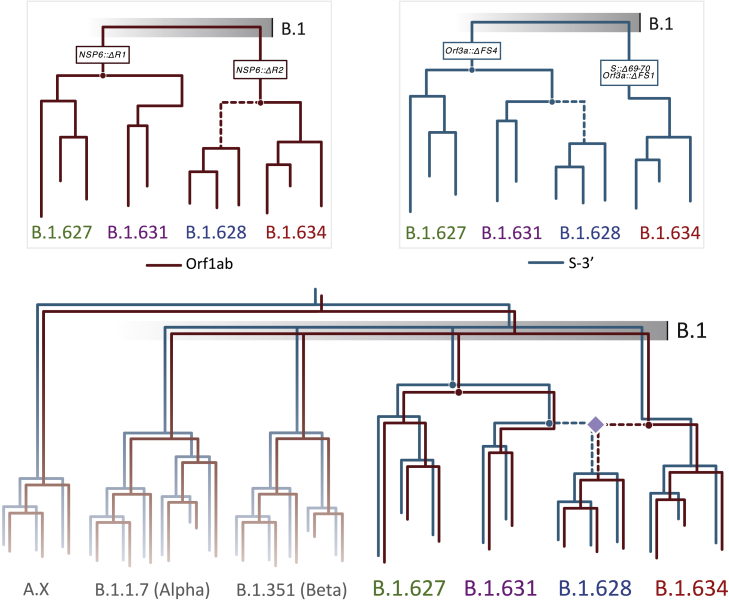
Schematic of the emergence of lineages B.1.627, B.1.628 major, B.1.631 major, and B.1.634 from a B.1 background and their recombination history The evolutionary trajectories of both genome segments for the four lineages (excluding the *minor* lineages) require a single occurrence of each of the deletions in ΔNSP6 and ΔOrf3a to explain most parsimoniously the observed deletion patterns (upper). The recombination history that reconciles these deletions while maintaining the inferred ancestors through phylogenetic analyses requires the occurrence of one recombination event, where lineages B.1.631 major and B.1.634 result in the emergence of a recombinant B.1.628 major lineage (lower). The dashed lines show tree branches that lead to the differing evolutionary trajectories of the two genome segments of B.1.628, where the recombination event that led to its emergence (purple diamond) likely took place. B.1.627 is evolutionarily related to B.1.631 major but not involved in the recombination event.

**Figure 5:**
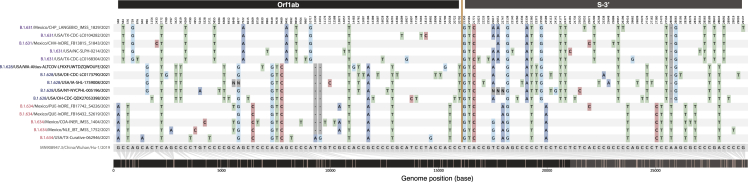
Comparison of single-nt polymorphisms (SNPs) between the B.1.631, B.1.628, and B.1.634 lineages SNPs were identified in reference to the 2019 Wuhan-Hu-1 reference genome (MN908947.3), shown in gray in the bottom line. Five sequences from the putative parental B.1.631 major lineage are shown on the top (purple), followed by five sequences from the putative recombinant B.1.628 major lineage in the middle (blue), and five sequences from the putative parental B.1.634 lineage at the bottom (red). The recombination point relative to the SNPs is marked by the yellow line and by different shading colors in the genome position bar (bottom).

Mapping the deletions to a phylogenetic tree inferred for the whole genome and for the complete dataset results in these appearing as homoplasic events that require repeated occurrence; specifically, FS4 would have had to occur twice (once in the branch leading to B.1.627 and B.1.631 major and once in B.1.628 major; Figure S4). Therefore, the most parsimonious model that reconciles the minimum required number of deletions and the phylogenetic incongruencies between the evolutionary histories of both non-recombinant genome segments results in B.1.628 major having descended from a recombination event. It inherited the Orf1ab segment (carrying the ΔR1 deletion on NSP6) from the lineage leading to B.1.634 and the S-3′ segment (carrying the FS4 deletion on Orf3a) from the lineage leading to B.1.631 major (Figure 4). Visualizing the SNPs of these lineages shows that B.1.628 major shares at least 6 polymorphisms with B.1.631 major in the first ∼17,000 nt of the genome and at least 9 polymorphisms with B.1.634 in the final ∼8,000 nt of the genome—no polymorphisms are shared between B.1.628 major and B.1.631 major along the 3′ end of the genome, whereas no polymorphisms are shared between B.1.628 major and B.1.634 along the 5′ end (Figure 5).

## Discussion

Genomic recombination has been widely described across sarbecoviruses in general (Boni et al., 2020) and has been identified as an important driver in the evolution of the lineage leading to the emergence of SARS-CoV-2 (Li et al., 2020; Lytras et al., 2021). Recombination between the B.1.1.7 and B.1.177 lineages has been observed in the United Kingdom leading to a limited number of circulating genomes and to their designation as lineage XA, the first recombinant SARS-CoV-2 lineage under the Pango nomenclature (Jackson et al., 2021). However, at the time of writing, no major circulating recombinant lineages spanning wider spatiotemporal distributions—across multiple countries—had been described. In this work, we investigate the evolutionary histories of four distinct but unusually similar SARS-CoV-2 Pango lineages circulating predominantly in the USA and Mexico and test the hypothesis that a recombination event led to the emergence of at least one of these lineages. A model can be proposed that resolves the phylogenetic incongruencies and deletion events among these lineages in which B.1.628 major originated from a single recombination event (Figure 4). The early identification of this lineage in the USA and Mexico and its widespread circulation elsewhere represents a notable event in the COVID-19 pandemic, as no previously recognized recombinant lineages have been reported to spread across country borders and to increase in frequency at the rate observed here. Following our results, the Pango Network committee decided that lineage B.1.628 *major* would be designated as lineage XB, making it the second recombinant lineage in the nomenclature system.

A necessary condition for the emergence of recombinant viruses is the co-circulation of its parental lineages (Boni et al., 2008, 2010), as viral recombination necessarily occurs during co-infection events of a single host (Simon-Loriere and Holmes, 2011). This condition is generally observed for the four lineages under investigation, predominantly detected in the USA and Mexico (where overlapping temporal distributions suggest co-circulation), as well as in other countries in Central America (Figure 1; Table 1). The substantial number of sequences and spatiotemporal distribution of B.1.628 major might normally be interpreted as evidence of an earlier emergence compared with B.1.627, B.1.631, and B.1.634. However, sampling intensity and the relative frequency of different lineages in the region require careful consideration (Kraemer et al., 2021a), particularly given the considerable disparities in sampling intensity in the context of genomic surveillance (Brito et al., 2021).

B.1.627 and B.1.628 major were the first lineages to be detected and were particularly frequent in the USA (among these four lineages). Both increased in frequency in the USA between January and May 2021, giving a reasonably strong indication that the recombination event occurred during this time (Figure 1A). Although the detection of B.1.627 declined to low levels by May, B.1.628 exhibited a second peak in detection in the USA in July 2021 (Figure 1; Figure S5A). Mexico reported sequences, predominantly of B.1.628 major and B.1.634, from May to July (Figure 1; Figure S5B), coinciding with the start of a systematic, nationwide genomic surveillance program under the CoVi-Gen Mex Consortium on May 11, 2021 (CONACYT, 2021). Given the differences in genome sampling and sequencing intensity between the two countries during the months preceding the detection of these lineages (6.3% of confirmed cases were sequenced in the USA during the last week of March 2021, compared with 1.6% for Mexico; Brito et al., 2021), it is likely that early cases of the B.1.628 major were not detected by genomic surveillance. However, the regional distribution of the lineages does suggest the recombinant lineage emerged in North America between late 2020 and early 2021.

Both minor clusters identified for B.1.628 and B.1.631 fail to display the monophyly condition which defines the Pango nomenclature (Rambaut et al., 2020). The phylogenetically distinct B.1.631 minor cluster was exclusively sampled in Turkey between late June and early August. Its spatiotemporal features and phylogenetic placement (Figure 3) and its distinct genome-wide divergence to the reference genome (Figure S2) warrants further investigation. However, its relevance in the evolution of the remaining lineages appears inconsequential.

Existing methods for inferring recombination events from genomic data are based on detecting clustering patterns for substitutions along sequences, measuring divergence (or genetic distance) across lineages, and testing for phylogenetic congruency (Posada and Crandall, 2001). Thus, the accurate detection of recombination breakpoints depends on the level of divergence between sequences/lineages and on the robustness of the phylogenies tested. Another limitation of these methods is that they can interpret high degrees of homoplasy as a potential signal of recombination. Given the overall genetic similarity and short divergence between the different SARS-CoV-2 lineages studied here (and in general), it is difficult to accurately estimate specific breakpoint positions with confidence (De Maio et al., 2020). This is why the exact location of the breakpoint we inferred differed depending on the dataset and methods used. However, all inferred breakpoints fall within the same genomic region corresponding to the end of Orf1ab and the beginning of the S gene reading frame. In either case, recombination analyses result in the placement of the NSP6 deletions on one side of the breakpoint and the Orf3a deletions on the other side. The mapping of these deletions provides an entirely independent data source to evaluate the occurrence of recombination from the commonly used nucleotide-level sequence changes that were also used to test for genomic mosaicism—the consistency between the results from these two data types provides strong evidence for the occurrence of a recombination event in our data. Furthermore, an inferred recombination breakpoint located near the start of the S protein reading frame generally coincides with previously described recombination hotspots for other coronaviruses (de Klerk et al., 2021; Sabir et al., 2016; Yang et al., 2021) and for SARS-CoV-2 (Boni et al., 2020; Jackson et al., 2021; Li et al., 2020; Lytras et al., 2021).

The two inferred GARD breakpoints from our datasets (i.e., leading to lineage XB) are biologically relevant, located on the S protein signal peptide or on the RBD. A breakpoint on the S protein signal peptide would produce a viral genome in which each reading frame is inherited from one of the two parental lines in its entirety, whereas a breakpoint on the RBD would result in a chimeric S protein. This latter possibility remains plausible given that the breakpoint would not disrupt major functional features of the protein (such as the trimeric ACE2-binding interface) and that sequence divergence remains low between these closely related lineages. Another interesting observation is that the canonical sarbecovirus transcription regulatory sequence (TRS), a 7-nt sequence that regulates the viral protein expression during cell infection (Yang et al., 2021), is located 18 nt upstream of the GARD breakpoint obtained with the full dataset. The TRS is also associated with viral genome replication, providing a possible mechanism driving recombination at this particular breakpoint (Yang et al., 2021). From an inferential standpoint, given the uncertainty regarding the precise location of the recombination breakpoint, exploring the individual phylogenetic tree of Orf1ab independently from the phylogeny for the remainder of the genome should adequately explain the complete evolutionary history of the lineages under investigation. This is particularly important given that RDP4 analyses show a recombination breakpoint upstream of the sites identified by GARD but still downstream of the NSP6 deletion.

Genetic recombination can have important consequences for viral adaptation and fitness, and it has been observed in many other viruses. Although mechanistically distinct from SARS-CoV-2, recombination in HIV has led to the emergence of successful circulating recombinant forms (CRFs) associated with high prevalence in some locations (Hemelaar et al., 2006; Vuilleumier and Bonhoeffer, 2015), and it has been hypothesized that enhanced replication-associated fitness may be involved (Njai et al., 2006). A hepatitis C virus (HCV) CRF was identified in St Petersburg, Russia (Kalinina et al., 2002) and has circulated for a prolonged time and across multiple countries (Raghwani et al., 2012). The seasonal human coronaviruses (HCoVs) 229E, HKU1, NL63, and OC43 show evidence of frequent recombination among individual genome sequences and occasionally among entire clades—recombinant monophyletic clusters have been described for HCoV-OC43 and HCoV-NL63, for example (Pollett et al., 2021). This pattern extends beyond the human coronaviruses. Lineage 5 of the zoonotic MERS-CoV shows evidence of having emerged through recombination and was associated with multiple human cases in Saudi Arabia and South Korea in 2014, as well as with camel infections (Sabir et al., 2016).

It is possible that the widespread circulation of the B.1.628 major lineage was in part a consequence of the effects of recombination on virus fitness. However, there is no direct support for this hypothesis and the expansion of any given lineage is likely driven by a myriad of factors in addition to virus genetics (Kraemer et al., 2021b). The persistence and spread of B.1.628 major means that, at the very least, recombination had no detrimental effects on its fitness. Recombination can increase viral genetic diversity by bringing together new combinations of circulating mutations into a single genome or haplotype—this can potentially purge deleterious mutations and overcome clonal interference (Simon-Loriere and Holmes, 2011). Through this mechanism, viruses can achieve large “jumps” in sequence space without requiring the generation of intermediate forms through cumulative mutation. This is of particular importance if these intermediate forms are selectively deleterious (Moradigaravand and Engelstädter, 2012); under such circumstances, recombination can enable virus species to jump from one fitness peak to another across a valley in the fitness landscape (Crona, 2018). Exploring the extent to which recombination can drive adaptation in SARS-CoV-2 and other human coronaviruses is paramount to evaluating the long-term effects on virus evolution.

Although our model for the recombinant origin of B.1.628 major reconciles the deletion and phylogenetic patterns observed in the genomic data, it still does not resolve all differences between the tree topologies for the Orf1ab and S-3′ regions, for example, individual sequences with no deletions are occasionally found in clusters/lineage that are characterized by those deletions. If these sequences are correct, then that would imply repeated reversion of the deletion—this is thought to be highly unlikely as there are no known mechanisms by which a specific combination of nucleotides (i.e., the ancestral sequence) would be inserted into a site where a deletion previously occurred. The insertion of predictable short sequences has only been generally described for specific genetic elements (Sehn, 2015). We, therefore, conclude that these apparent reversions are more likely artifacts deriving from sequencing or assembly errors—this would be consistent with the presence of genome sequences in our dataset where the deletion site falls among highly ambiguous positions (De Maio et al., 2020).

We conclude that the B.1.628 major lineage arose from a recombination event between B.1.631 major and lineage B.1.634, prompting its designation as a recombinant lineage under the Pango nomenclature. We also note that the group of sequences identified here as B.1.628 has been revisited and re-designated as lineage B.1. The expansion of B.1.628 during November 2020 and March 2021 coincided with a peak in the average number of daily confirmed COVID-19 cases recorded in North, Central, and South America (Ritchie et al., 2020). However, at least in Mexico and in the USA, none of the lineages studied here reached a detection frequency of >1% relative to other co-circulating lineages within the region at that given timeframe (CONACYT, 2021; Hodcroft, 2021). Nonetheless, the drastic sweep of the B.1.1.529 sublineage BA.1 (VOC Omicron) that was preceded by the temporary dominance of the lineage B.1.617.2 (VOC Delta) highlights the viability for new lineages to emerge and replace currently circulating variants. This process can potentially involve other recombinant SARS-CoV-2 lineages, delineating yet another key function of active genomic surveillance programs. Our findings also emphasize the importance of further investigating the recombination rate and potential of the virus and of exploring the drivers of such evolutionary processes.

## STAR★Methods

### Key resources table

**Table:** 

REAGENT or RESOURCE	SOURCE	IDENTIFIER
**Deposited data**		

Mapped deletions across virus genome sequences	Github repository	Table S1; https://github.com/BernardoGG/XB_lineage_investigation
Virus genome sequence data	Global Initiative on Sharing Avian Influenza Data	Table S2; https://www.gisaid.org
Daily number of reported COVID-19 deaths	Our World in Data	Figure 1; https://ourworldindata.org/coronavirus

**Software and algorithms**		

MAFFT v7.487	Katoh and Standley (2013)	https://mafft.cbrc.jp/alignment/software/
Sequence subsampling	This paper	https://github.com/BernardoGG/XB_lineage_investigation
IQ-TREE v2.1.3	Minh et al. (2020)	http://www.iqtree.org/
UShER	Turakhia et al. (2021)	https://github.com/yatisht/usher
FigTree v1.4.4	GitHub repository	https://github.com/rambaut/figtree/releases
GARD	Kosakovsky Pond et al. (2006)	https://www.datamonkey.org/gard
RDP4	Martin et al. (2015)	http://web.cbio.uct.ac.za/∼darren/rdp.html
*ape* R package	Paradis and Schliep (2019)	https://cran.r-project.org/web/packages/ape/ape.pdf
Pairwise genetic distances	This paper	Figure S2; https://github.com/BernardoGG/XB_lineage_investigation
snipit	GitHub repository	https://github.com/aineniamh/snipit

**Other**		

Sequence alignments, analysis outputs and acknowledgements list	This paper	https://github.com/BernardoGG/XB_lineage_investigation

### Resource Availability

#### Lead contact

Further information and requests for resources and reagents should be directed to and will be fulfilled by the lead contact, bernardo.gutierrez@zoo.ox.ac.uk.

#### Materials availability

This study did not generate new unique reagents.

### Method details

#### Identification of SARS-CoV-2 lineages with high degree of genetic similarity

We downloaded complete genome sequences from GISAID (Shu and McCauley, 2017) from individual Pango lineages detected in countries from Central America and Mexico (as of August 12 2021) and generated a consensus list of mutations for each lineage. This was done by extracting individual mutations per genome in relation to the SARS-CoV-2 reference genome Wuhan-Hu-1 (Wu et al., 2020) using the Augur pipeline (Hadfield et al., 2018) and identifying the ones that were found in >85% of the sequences assigned to said lineage. Each entry in this consensus mutation list identifies the locus, position (in reference to Wuhan-Hu-1) and type of nucleotide change. From these consensus mutation lists for each lineage, we generated a pairwise matrix of the number of shared mutations between individual lineages (i.e., absent in Wuhan-Hu-1 and shared by individual pairs of lineages). Lineages with unusually high numbers of shared mutations were visually identified further analysed as described below. The results from these preliminary analyses have been presented as a Twitter thread at https://twitter.com/shay_fleishon/status/1425775733167820814 and as a Github Issue at https://github.com/cov-lineages/pango-designation/issues/189.

To contextualize the epidemiological scenario under which these lineages emerged and circulated in the region, we retrieved the daily number of COVID-19 reported deaths between July 2020 and August 2021, aggregated across all countries in North America and Central America, from the Our World in Data repository (Ritchie et al., 2020).

#### Genomic data, metadata and sequence alignment

We retrieved all complete SARS-CoV-2 genome sequences assigned to Pango lineages B.1.627, B.1.628, B.1.631 and B.1.634 as of August 30 2021 from GISAID (Shu and McCauley, 2017). Accompanying sequence metadata, including sampling locations (at different geographic resolutions) and dates of sample collection and submission were also retrieved. This complete data set included 1950 sequences that were subsequently filtered to exclude all sequences for which >10% of sites were ambiguous (i.e., had nucleotide states N or X). The final data set, comprising 1055 complete genome sequences, was used for all phylogenetic analyses. The original complete data set (n=1950) was used in part to explore the spatio-temporal distribution of the four Pango lineages under investigation.

After adding the reference SARS-CoV-2 genome Wuhan-Hu-1 (GenBank accession MN908947.3; Wu et al, 2020) to the filtered data sets, the sequences were aligned using MAFFT v7.487 (Katoh and Standley, 2013). The resulting alignments were inspected visually to identify deletions >1nt in length and which were shared by all or most of the sequences assigned to one or more of the lineages under investigation; these deletions were removed from the alignment and encoded as discrete sequence traits, which were later mapped onto estimated phylogenetic trees (see results).

#### Confirmation of Pango lineage assignment and whole genome phylogenetic analysis

The Pango lineage assignment of the sequences in our final data set was determined using Pangolin v.3.1.11 (Rambaut et al., 2020); all lineages originally assigned on GISAID were confirmed. To further explore the phylogenetic structure of our data, we constructed a maximum likelihood (ML) phylogenetic tree using IQ-TREE v2.1.3 (Minh et al., 2020) under a GTR+Γ substitution model. We estimated node support values using the Shimodaira-Hasegawa (SH) approximate Likelihood Ratio test (SH-aLRT; Guindon et al., 2010), with 1000 replicates, and 1000 bootstrap replicates. Given that it has been recently suggested that UShER (Ultrafast Sample placement on Existing tRees; Turakhia et al., 2021) presents an increased lineage assignment stability compared to PangoLEARN (Schneider et al., 2022), we performed an additional phylogenetic reconstruction of the four lineages in UShER using a 200-sequence subsampled data set that was phylogenetically placed into a 2000-sequence background.

### Quantification and statistical analysis

#### Genome-wide divergence of Pango lineages and recombination breakpoint inference

Pairwise genetic distances across the length of the genome of basal sequences for each of the lineages under investigation and the reference Wuhan-Hu-1 genome were estimated using custom scripts (available at https://github.com/BernardoGG/XB_lineage_investigation), based on the *ape* package in R (Paradis and Schliep, 2019). One sequence was selected per lineage, and in cases where multiple important clades were identified within single Pango lineage one basal sequence from each of these clades was included. Raw genome-wide distances were estimated for 500-nucleotide segments, and a sliding window approach was used to estimate these distances across segments that overlapped every 20 nucleotides.

Recombination tests are computationally demanding for large data sets. To address this, we further subsampled the alignment to include one sequence per country per Pango lineage per day (script available at https://github.com/BernardoGG/XB_lineage_investigation), resulting in a downsized set of 716 whole genome sequences. To evaluate recombination patterns in our data, we further reduced the downsized data set by randomly sampling 200 sequences from the four lineages under investigation. This reduced data set was then analysed using GARD (Kosakovsky Pond et al., 2006), a tool that uses a genetic algorithm (GA) to search for one or more putative breakpoints in a multiple sequence alignment. The best supported number of non-recombinant fragments in the alignment is then evaluated by comparing the Akaike Information Criterion (AIC_c_) of the proposed models versus a null model (i.e., no recombination points, such that a single tree topology best explains the sequence alignment). The resulting Δc-AIC estimates between the best supported model compared to the null model and to the “single tree multiple partition” model shows the statistical support enclosing all iterations of the GA with an evidence ratio of 100 or greater (Kosakovsky Pond et al., 2006). The phylogenetic topological incongruencies of potential non-recombinant fragments can be evaluated to identify lineages involved in the recombination event (Kosakovsky Pond et al., 2006). We then compared the results obtained from GARD with those obtained using other recombination detection methods with a different approach. For this, we ran RDP4 (Martin and Rybicki, 2000; Martin et al., 2015), a computer program that sequentially tests every combination of three sequences in an alignment to find evidence that one of the three sequences is a recombinant and the other two are parental. We analysed the aforementioned 716-sequence alignment using the methods implemented in RDP4: Bootscan, MaxChi, Chimaera, 3Seq, GENECONV, SiScan and RDP (Martin et al., 2015). Statistical tests are unique to each recombination detection method in RDP4, and approximate *p-value*s are reported where evidence for recombination was identified.

#### Phylogenetic analyses of inferred non-recombinant genome segments

Given the possibility that a single phylogeny of the complete genome alignment does not explain the evolutionary history of our sequences, we partitioned the alignment using the inferred breakpoints from GARD (derived from the analysis of the 200-sequence data set). ML trees for each genome partition were then estimated with IQ-TREE as described previously.

We identified four key deletions amongst our sequences under investigation: two deletions in the NSP6 gene (Orf1ab) and two deletions in Orf3a (see results). NSP6 deletions occur at two adjacent locations (here called ΔR1 and ΔR2) and don’t result in changes in the reading frame. Orf3a deletions occur on a single locus and take the form of either a single-nucleotide frameshift deletion (ΔFS2) or a 4-nucleotide frameshift deletion (ΔFS4). These deletions were coded as discrete characters (Table S1), assigned to individual tree tips and reconstructed at internal nodes using a parsimony criterion, thereby visualising the history of their occurrence across the phylogenies; these patterns have to reconcile with the proposed recombination events. Our rationale is that the individual evolutionary histories of each of the putative non-recombinant fragments should also parsimoniously explain the occurrence of deletions observed in NSP6, S and ORF3a, under the assumption that deletions do not revert once they occur in a lineage. Our rationale also draws from the premise that these deletions are more likely to descend from single occurrences within the evolution of each lineage but are not restricted to have occurred just once across the whole phylogeny. Specifically, some of these deletions have been observed previously in other lineages and variants of concern, including the NSP6 deletions observed in B.1.1.7, P.1 and B.1.351 (Meng et al., 2021). The genomic position where a given deletion occurs (relative to the inferred recombination breakpoints) was used to determine which genome partition most likely represents the true evolutionary history of that deletion. Loci where the deletion was flanked by ambiguities were differentially labelled with an asterisk (^∗^).

#### Exploring the phylogenetic discrepancies of the lineages under investigation relative to other B.1 lineages

Our phylogenetic analyses showed that lineages B.1.628 and B.1.631 are split into two groups each. B.1.628 contains a cluster of sequences that fall near the root of the phylogenies and are henceforth identified as *B.1.628 minor*, and a large more derived monophyletic clade henceforth identified as *B.1.628 major*. B.1.631 is split into a small cluster of sequences that consistently cluster near the tree backbone in both genome segments and is henceforth identified as *B.1.631 minor*, and a larger monophyletic clade here called *B.1.631 major*. To explore the topological discrepancies between the lineages under investigation in the absence of B.1.631 minor (see results for an explanation of why this cluster was excluded), we randomly sampled five sequences from each of the lineages under investigation (B.1.627, B.1.628 major, B.1.628 minor, B.1.631 major and B.1.634), with five random sequences from the B.1.1.7 lineage (i.e. VOC Alpha) and five random sequences from the B.1.351 lineage (i.e. VOC Beta). This approach was used in order to explore the congruency of the diversification patterns of the lineages under investigation in context of the B.1 lineage. Sequences from the Alpha and Beta VOCs were chosen solely as a reference for widely sampled outgroups and were chosen because (i) they are distinct monophyletic lineages, and (ii) they circulated widely during the time period corresponding to this investigation. Five additional random sequences from lineage A.2.5 were also included to represent the Pango A lineage and to provide an outgroup for tree rooting. All sequences from the B.1.1.7, B.1.351 and A.2.5 lineages were obtained from GISAID; they had sampling dates that spanned the time when these lineages were observed to circulate and were predominantly from locations in North and Central America. We performed GARD analyses and constructed ML phylogenetic trees from this data set as previously described. We estimated node support for the phylogenetic analyses using 1000 bootstrap replicates, and nodes with >50% node support were collapsed into polytomies.

Finally, we used the snipit software (https://github.com/aineniamh/snipit) to explore the distribution of single nucleotide polymorphisms (SNPs) across the genome of potential recombinant and parental lineages. SNPs were identified and visualised in reference to the Wuhan Hu-1 genome sequence.

## Data Availability

• SARS-CoV-2 genome sequences used in this study were retrieved from the Global Initiative on Sharing Avian Influenza Data repository (GISAID) at https://www.gisaid.org. Epidemiological data on daily reported COVID-19 cases for North America, Central America and the Caribbean were obtained from Our World in Data at https://ourworldindata.org/coronavirus. • All the code generated and used for this study is publicly available on Github at https://github.com/BernardoGG/XB_lineage_investigation. • Any additional information required to reanalyze the data reported in this paper is available from the Lead Contact upon request.

## Supplemental information

Document S1. Figures S1–S5

Table S1. Mapped re-occurring deletions in the SARS-CoV-2 genome across the sequences used in this study, coded as discrete characters, related to Figure 3 Tab delimited file version available at https://github.com/BernardoGG/XB_lineage_investigation/blob/main/Data/XB_analysis_deletion_mapping.tsv.

Table S2. List of laboratories and authors generating genome sequence data used in this study, publicly shared through the GISAID platform, related to Figures 1 and 3 Tab delimited file version available at https://github.com/BernardoGG/XB_lineage_investigation/blob/main/Acknowledgements/xb_20210831_acknowledgements.tsv.

Document S2. Article plus supplemental information
