# Supplementary material for: Emergence and widespread circulation of a recombinant SARS-CoV-2 lineage in North America
Source: Cell Host Microbe. 2022 Aug 10;30(8):1112–1123.e3. doi: 10.1016/j.chom.2022.06.010 (PMC9212848; doi:10.1016/j.chom.2022.06.010)
Supplement: Document S1. Figures S1–S5 [file mmc1.pdf]

Cell Host & Microbe, Volume *30*

Supplemental information

Emergence and widespread circulation of a recombinant SARS-CoV-2 lineage in North America

Bernardo Gutierrez, Hugo G. Castelán Sánchez, Darlan da Silva Candido, Ben Jackson, Shay Fleishon, Renaud Houzet, Christopher Ruis, Luis Delaye, Nuno R. Faria, Andrew Rambaut, Oliver G. Pybus, and Marina Escalera-Zamudio

Emergence and widespread circulation of a recombinant SARS-CoV-2 lineage in North America

Bernardo Gutierrez[1,2,3,15,*], Hugo G. Castelán Sánchez[2,4], Darlan da Silva Candido[1,5], Ben Jackson[6], Shay Fleishon[7], Renaud Houzet[8], Christopher Ruis[9,10], Luis Delaye[2,11], Nuno R. Faria[1,5,12,13] Andrew Rambaut[6], Oliver G. Pybus[1,14,*], Marina Escalera-Zamudio[1,2,*]

[1]*Department of Zoology, University of Oxford, UK*
[2]*Consorcio Mexicano de Vigilancia Genómica (CoViGen-Mex)*
[3]*Colegio de Ciencias Biológicas y Ambientales, Universidad San Francisco de Quito USFQ, Ecuador*
[4]*Consejo Nacional de Ciencia y Tecnología, Ciudad de México, México*
[5]*Instituto de Medicina Tropical, Faculdade de Medicina da Universidade de São Paulo, Brazil*
[6]*Institute of Evolutionary Biology, University of Edinburgh, UK*
[7]*Independent researcher; s.fleishon@gmail.com*
[8]*Independent researcher; renaud.houzet@hotmail.fr*
[9]*Molecular Immunity Unit, Department of Medicine, University of Cambridge, Cambridge, UK*
[10]*Department of Veterinary Medicine, University of Cambridge, Cambridge, UK*
[11]*Departamento de Ingeniería Genética, Unidad Irapuato, CINVESTAV, Mexico*
[12]*MRC Centre for Global Infectious Disease Analysis, School of Public Health, Imperial College London, London, UK*
[13]*The Abdul Latif Jameel Institute for Disease and Emergency Analytics, School of Public Health, Imperial College London, London, UK*
[14]*Department of Pathobiology, Royal Veterinary College, London UK*
[15]*Lead contact*

Supplemental Information

Supplemental figures S1 – S5

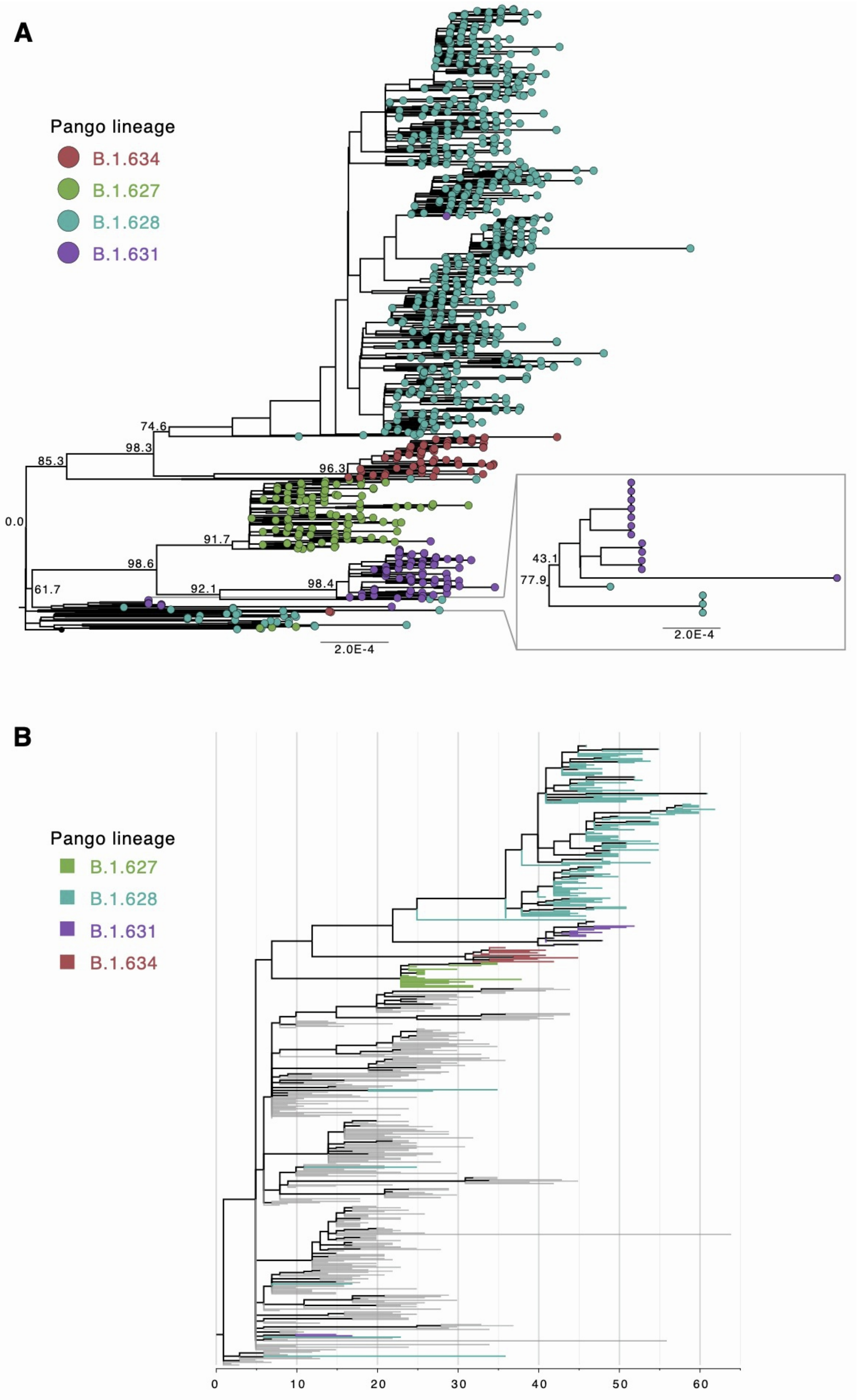

Figure S1. Maximum likelihood (ML) phylogenetic analyses of the complete SARS-CoV-2 genome for the four lineages, related to Figure 3. **(A)** ML tree generated in IQTree. The individually designated PANGO lineage for each sequence is highlighted (the predominant lineage for sections of the tree shown), and SH-aLRT node support is shown for key lineage defining nodes on both phylogenies. **(B)** Phylogenetic placement of 200 sequences from the four lineages on a 2000-sequence background data set with UShER.

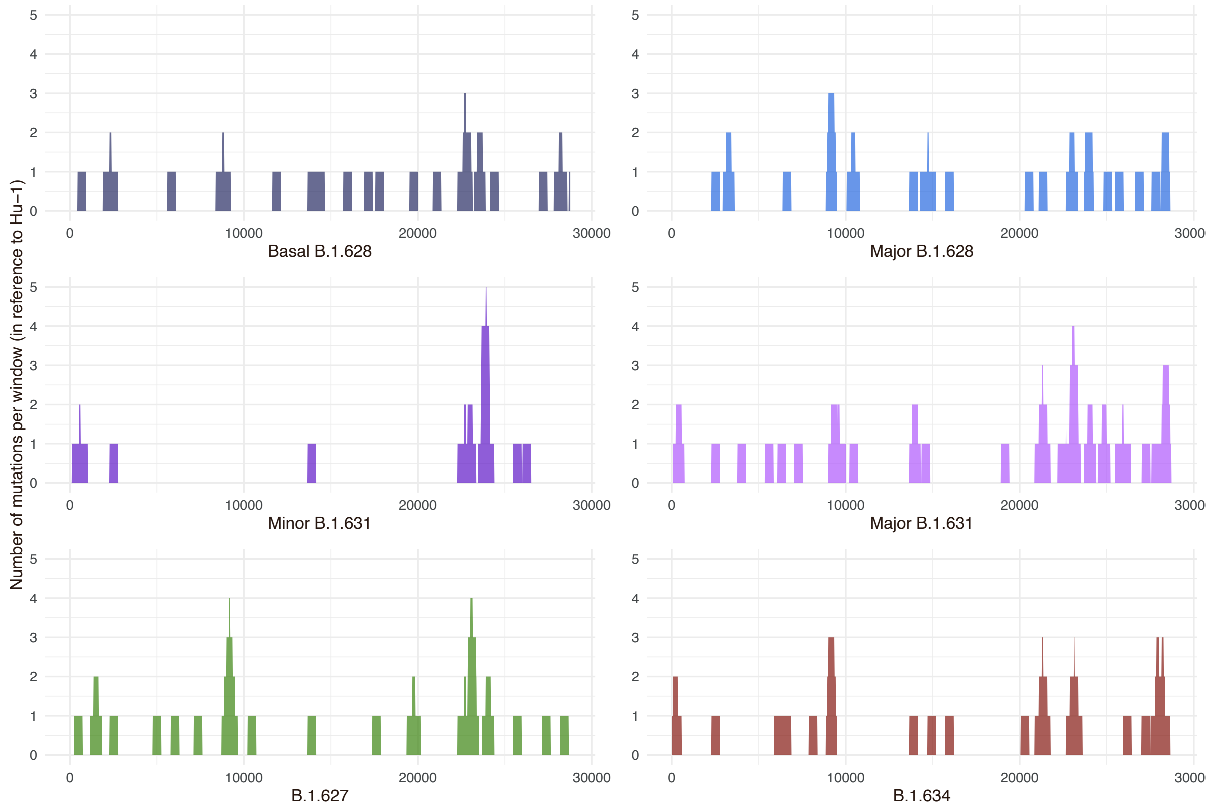

Figure S2. Genetic distance plots across the SARS-CoV-2 genome between the main SARS-CoV-2 phylogenetic clusters amongst the four lineages under investigation and the reference Wuhan-Hu-1, related to Figure 3. The number of mutations between basal sequences for each of the monophyletic groups identified in the phylogenetic analyses and the 2019 Wuhan-Hu-1 reference genome (MN908947.3). Mutation numbers were estimated from genomic segments of 500 nucleotides in length, overlapping over 20-nucleotide intervals.

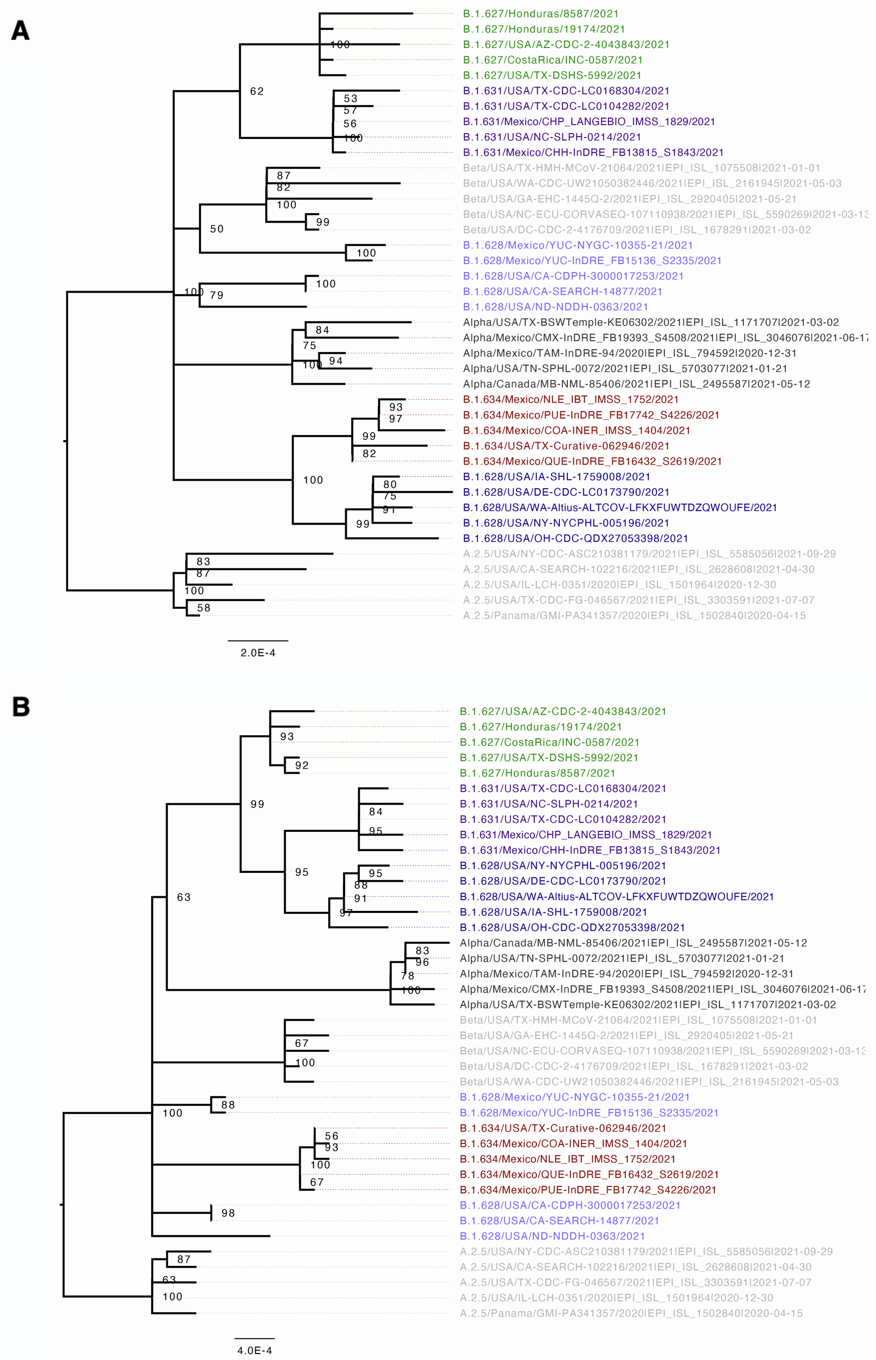

Figure S3. Maximum likelihood phylogenetic tree for the Orf1ab **(A)** and S-3' **(B)** genome segments of a selection of sequences of lineages under investigation B.1.627 (green), B.1.628 minor (light blue), B.1.628 major (dark blue), B.1.631 (purple) and B.1.634 (red) in relation to B.1.1.7 (VOC Alpha, dark grey) and B.1.351 (VOC Beta, fark grey), related to Figure 5. Node support is shown from 1000 bootstrap replicates, nodes with support <50% are collapsed into polytomies. The tree is rooted in reference to lineage A.2.5.

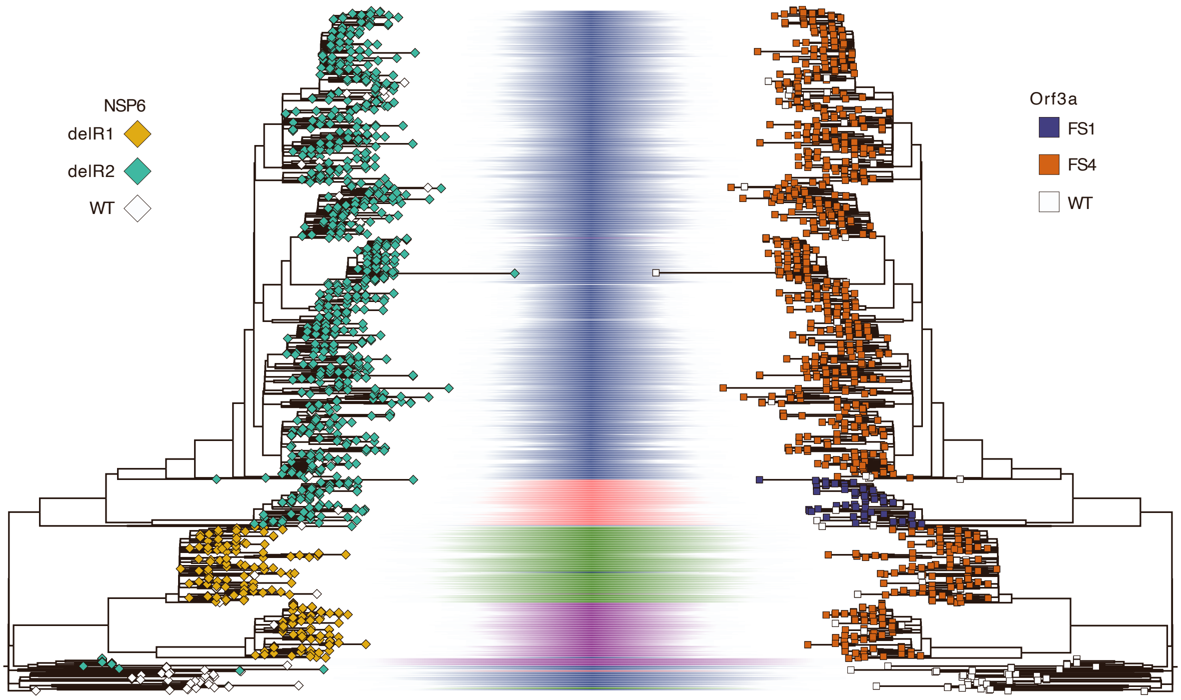

Figure S4. Deletions on the Orf1ab (NSP6; diamonds) and Orf3a (rectangles) loci mapped to the maximum likelihood phylogeny of the complete genome for the four lineages under investigation, related to Figure 3. Major lineage designations are shown in coloured shading: B.1.627 (green), B.1.628 (blue), B.1.631 (purple) and B.1.634 (red).

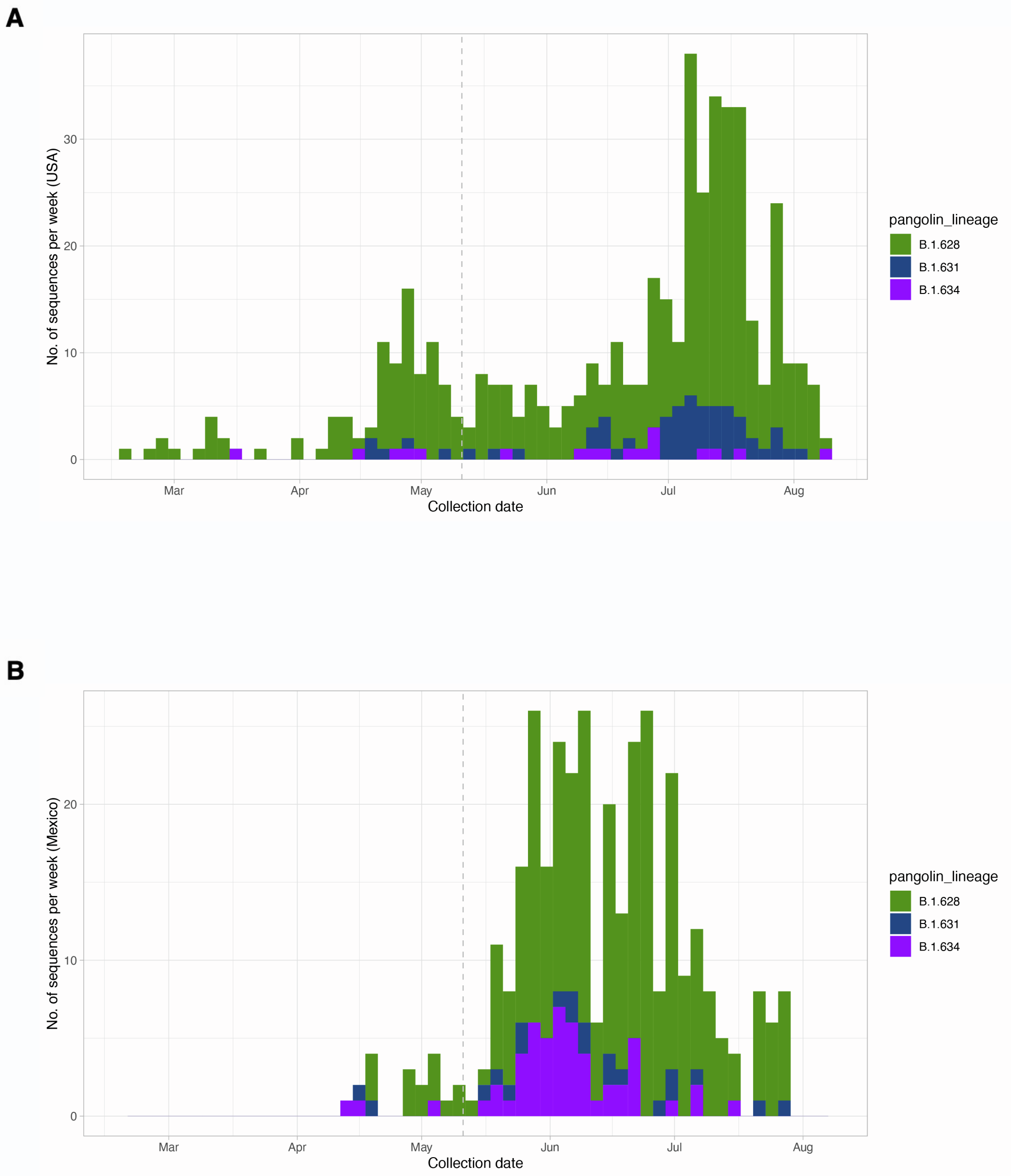

Figure S5. Sequence sampling from the B.1.628 major, B.1.631 and B.1.634 lineages in the USA **(A)** and Mexico **(B)**, related to Figure 1. Sequences shown here correspond only to the sequences included in the phylogenetic analyses (i.e. <10% ambiguities in the genome sequence, >90% genome coverage). Dotted line shows the starting date for the systematic genomic surveillance work performed in Mexico by the CoVi-Gen Mex Consortium.
